# Prevalence, severity, and predictors of symptom burden among adolescents and young adults with cancer

**DOI:** 10.1002/cam4.5837

**Published:** 2023-03-27

**Authors:** Sumit Gupta, Qing Li, Paul C. Nathan, Norma D'Agostino, Nancy N. Baxter, Colleen Fox, Karine Chalifour, Natalie Coburn, Rinku Sutradhar

**Affiliations:** ^1^ Division of Haematology/Oncology The Hospital for Sick Children Toronto Canada; ^2^ Cancer Research Program, ICES Toronto Canada; ^3^ Institute for Health Policy, Evaluation and Management, University of Toronto Toronto Canada; ^4^ Faculty of Medicine University of Toronto Toronto Canada; ^5^ Department of Supportive Care Princess Margaret Cancer Centre Toronto Canada; ^6^ Li Ka Shing Knowledge Institute, St. Michael's Hospital Toronto Canada; ^7^ Melbourne School of Population and Global Health, University of Melbourne Carlton Victoria Australia; ^8^ Cancer Clinical Programs, Ontario Health Toronto Canada; ^9^ Young Adult Cancer Canada St. John's Canada; ^10^ Department of Surgery Sunnybrook Health Sciences Centre Toronto Canada; ^11^ Department of Surgery University of Toronto Toronto Canada; ^12^ Sunnybrook Research Institute Toronto Canada; ^13^ Dalla Lana School of Public Health, University of Toronto Toronto Canada

**Keywords:** adolescents, cancer, patient‐reported outcomes, population‐based, symptoms, young adults

## Abstract

**Background:**

Symptom burden in adolescents and young adults (AYA) with cancer is poorly characterized but impacts quality of life.

**Methods:**

All Ontario, Canada AYA aged 15–29 years at diagnosis between 2010 and 2018 were linked to population‐based healthcare databases, including to Edmonton Symptom Assessment System‐revised (ESAS) scores, an 11‐point scale routinely obtained at the time of cancer‐related outpatient visits and collected provincially. Multistate models estimated mean duration of symptom severity states [none (0) vs. mild (1 vs. 2 vs. 3) vs. moderate (4–6) vs. severe (7–10)], trajectories, and subsequent mortality risk. Variables associated with severe symptoms were also determined.

**Results:**

In total, 4296 AYA with ≥1 ESAS score within a year of diagnosis were included (median age 25 years). Prevalent moderate/severe symptoms included fatigue (59% of AYA) and anxiety (44%). Across symptom type, AYA reporting moderate symptoms were likelier to subsequently experience improvement versus worsening. Risk of death within 6 months increased with increasing symptom burden and was highest in AYA with severe dyspnea (9.0%), pain (8.0%), or drowsiness (7.5%). AYA in the poorest urban neighborhoods were more likely to experience severe symptoms than in the wealthiest areas, with twice the odds of reporting severe depression [adjusted odds ratio (OR) 1.95, 95th confidence interval (95% CI) 1.37–2.78], pain (OR: 1.94, 95% CI: 1.39–2.70), and dyspnea (OR: 1.96, 95% CI: 1.27–3.02).

**Conclusions:**

AYA with cancer experience substantial symptom burden. Risk of death increased with symptom severity. Interventions targeting cancer fatigue and anxiety, and targeting AYA in lower‐income neighborhoods, are likely to improve quality of life in this population.

## Graphical abstract

Proportion of ESAS ≥7 at each biweekly interval.

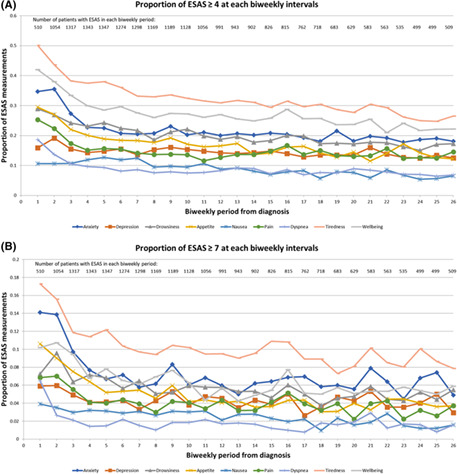

## 1 INTRODUCTION

Cancer patients experience significant symptom burden from the cancer, its treatment, and their long‐term consequences.^1^, ^2^ Inadequate symptom control is common and impacts quality of life.^3^, ^4^, ^5^, ^6^ Adolescents and young adults (AYA, often defined as between 15 and either 29 or 39 years) with cancer are an at risk subpopulation that experiences disparities in various outcomes.^7^, ^8^, ^9^ Literature examining symptom burden in AYA with cancer is sparse. A review focused on adolescents noted that the most common symptoms included fatigue, nausea, pain, and mood disturbances; over 50% of adolescents reported symptoms.^10^ Most of the 12 studies identified were assessed as “very low quality”; no study was rated higher than “low quality”. Sample sizes ranging from 8 to 220. Recent literature has suggested that AYA are more likely to experience cancer‐related symptoms, which are slower to resolve than in other age groups.^11^, ^12^

Two other important limitations exist in our knowledge of symptom burden in this population. First, symptom burden has traditionally been determined by healthcare providers. Systematic use of patient‐reported outcome measures is however more accurate, comprehensive, and when paired with interventions, improves quality of life and clinical outcomes.^13^, ^14^, ^15^, ^16^, ^17^, ^18^ Second, studies of symptoms among AYA with cancer have generally been cross‐sectional in nature and thus are unable to provide information on how symptoms evolve or resolve over time.

To overcome the above limitations, we leveraged healthcare databases in Ontario, Canada, including ones containing longitudinal patient‐reported symptom assessments, to build a population‐based cohort of AYA with cancer. We had two main objectives. First, we employed a multistate transition model to determine the prevalence, severity, and trajectory of symptoms in the cohort. Second, we used a logistic regression model to identify predictors of high symptom burden.

## 2 METHODS

### 2.1 Study setting

Canadian healthcare is delivered provincially through universal government health insurance plans. Adult care is delivered through Regional Cancer Centers (RCCs) and non‐RCC hospitals. Though individual hospitals may have AYA‐specific programs, no provincial AYA cancer care system exists. Individual AYA may receive care across multiple institutions.

The Edmonton Symptom Assessment System‐revised (ESAS) is a valid and reliable patient‐reported outcome measure assessing the presence and severity of nine common cancer‐associated symptoms: pain, tiredness, drowsiness, nausea, lack of appetite, shortness of breath, depression, anxiety, and overall well‐being.^19^, ^20^ Each is rated on an 11‐point scale: 0 (no symptoms) –10 (worst possible symptoms) (Appendix A). Scores are commonly categorized as: no symptoms (0), mild (1–3), moderate (4–6), and severe (7–10).^21^, ^22^

In 2007, Ontario‐implemented ESAS screening of cancer patients at adult hospitals at each cancer‐related outpatient visit (on or off therapy) to improve supportive care and achieve symptom control. ESAS screening was broadly available at all 14 RCCs by 2010, with implementation common but variable in non‐RCC hospitals.^23^, ^24^, ^25^ Web‐based platforms are used to collect ESAS data, with screening administered using kiosks, tablets, manual input of paper forms, or online completion. Multiple language options are available. Inpatients and patients supported at home through palliative care are not ESAS screened. All scores are collected centrally in the Symptom Management Reporting Database (SMRD). Additional details have been published.^23^, ^24^, ^25^ Though ESAS screening is for the most part offered to patients at each cancer‐related outpatient visit, patients may choose not to complete ESAS questionnaires.

### 2.2 Study population and data sources

We conducted a retrospective population‐based cohort study. All AYA aged 15–29 years at diagnosis of primary cancer between January 2010 and June 2018 were identified using the Ontario Cancer Registry. Patients were linked deterministically to population‐based health services databases housed at ICES, a research institute that holds an array of Ontario health‐related data, using unique encoded identifiers. The Activity Level Reporting (ALR) database contains data pertaining to patient level activity within the cancer system, mainly focused on outpatient oncology clinic visits, radiation, and systemic therapies. Centers must report patient‐level cancer activity to ALR. Other databases identified physician claims, services, and hospitalizations (Table S1).

Populations who did not have access to ESAS were excluded: (1) diagnosis and treatment in pediatric institutions; (2) did not require services at a cancer center in the first year after diagnosis (e.g., surgically resected thyroid cancer, removal of colonic polyps), and; (3) the small number of AYA who were treated exclusively at non‐RCCs.^26^ Finally, AYA who were not screened with ESAS during the first year after their cancer diagnosis were excluded, as no descriptions of their symptom burden could be made.

### 2.3 Symptom prevalence, severity, and trajectory

Patient ESAS scores were organized and analyzed using a Markov multistate transition model with seven states (Figure S1). Six non‐absorbing mutually exclusive states were defined corresponding to symptom absence (ESAS score 0), each of the mild scores as its own state (1–3, and), moderate scores (4–6), and severe scores (7–9). An absorbing state of death was also defined. Scores corresponding to mild severity (1–3, and) were treated as individual states based on the clinical impression that transitions within the mild categorization (e.g., 1–3) were clinically significant. Transitions were only permitted between consecutive non‐absorbing states in either direction (e.g., moderate to severe, or severe to moderate), or from any non‐absorbing state to death. Thus, patients with sequential ESAS scores corresponding to non‐consecutive states were assumed to have transitioned through all intermediate states between assessments (e.g., a patient with observed sequential scores of 1 and 3 was assumed to have passed through a score of 2 between these two assessments, whether gradually or instantaneously).^27^, ^28^ Multistate transition models account for intermittent observation (i.e., transitions between states in reality occurred at some point between consecutive assessments) and thus different intervals between ESAS measurements, as well as interval censoring.^29^

Patients were followed from diagnosis until the first of either death or the end of the follow‐up period (December 31, 2020). For each symptom state, the multistate model estimated the average length of time spent in that state (i.e., mean sojourn time), the relative rate of moving to a more vs. less severe symptom state by comparing instantaneous rates of transition, and the probability of death within 1 and 6 months. Patients with no ESAS score recorded within 2 months before or after their cancer diagnosis were assumed to have an initial ESAS score of 0. Sensitivity analyses were conducted assuming all patients' initial ESAS state was 0, or that excluded patients with only one ESAS score recorded during the first year of treatment.

### 2.4 Predictors of high symptom burden

To determine predictors of high symptom burden, the first year after diagnosis was divided into 2‐week periods and the maximum ESAS score for each symptom identified within each period. For each patient, periods without an ESAS assessment were excluded. The unit of analysis was thus each patient‐period; a patient with at least one ESAS score in each 2‐week period would thus contribute 26 observations (the maximum ESAS score in each period).

For each symptom, our two outcomes were: (1) whether the patient‐period was associated with a moderate or severe score (≥4), or (2) whether the patient‐period was associated with a severe score (score ≥7).

Potential predictors included demographic, disease, and treatment‐related variables. Demographic variables included age (continuous) and sex. Neighborhood income quintile and urban/rural status [rural vs. urban quintile 1 (poorest) through urban quintile 2 (richest)] was determined from census data and postal code at diagnosis.^30^, ^31^ Region was defined using the five main Ontario health regions (Central, East, North, Toronto, and West). Cancer type was categorized as hematologic, melanoma, central nervous system (CNS), sarcoma, testicular/ovarian, breast, colorectal, thyroid, and other. Initial cancer stage and disease status at the time of ESAS measurement were not consistently available. Time period of diagnosis was defined as early (2010–2014) and late (2015–2018). For each 2‐week period, institution type (RCC vs. non‐RCC) was defined as the institution visited most often in the 30 days prior to the start of the period. If no ALR visits occurred in the prior 30 days, the first institution visited during the 2‐week period was used.

Treatment‐related variables included receipt of chemotherapy, radiation, or surgery. Patients who in the previous 2 weeks had at least one ALR visit record indicating systemic therapy and noting a chemotherapy regimen or antineoplastic agent were defined as having received chemotherapy. Patients with ≥1 ALR record indicating radiation in the prior 8 weeks were defined as having received radiation. A list of all surgical procedure codes recorded within 1 year of diagnosis for the whole cohort was assembled, and manually categorized into cancer vs. non‐cancer surgeries by diagnostic category. Patients with a disease‐specific procedure code within the prior 8 weeks were considered to have had undergone cancer surgery. A counter variable representing the number of 2‐week periods elapsed since the cancer diagnosis was included.

Univariate and multivariable logistic regression models were constructed to determine whether potential predictors were associated with our two outcomes (score ≥4 and score ≥7). Generalized estimating equations accounted for correlation due to repeated measures from the same individual. Statistical significance was defined as *p* < 0.05. Analyses were performed using SAS Enterprise Guide, version 7.15 (SAS Institute). Ethics approval was obtained at The Hospital for Sick Children and Sunnybrook Health Sciences Centre. Informed consent was not required.

## 3 RESULTS

In total, 5435 AYA with cancer met inclusion criteria (Figure S2); 4296 (79.0%) had ≥1 ESAS score within a year of diagnosis. Baseline characteristics are shown in Table 1. Median time from cancer diagnosis to the first ALR visit was 24 days [interquartile range (IQR): 13–45]. The median number of ESAS assessments within first year was four (IQR: 2–9).

**TABLE 1:** Distributions of demographic and disease characteristics of study cohort (*N* = 4296).

	*N* (%)	Median (IQR)
Age at diagnosis (years)		
15–17	110 (2.6)	25 (22–27)
18–20	676 (15.7)	
21–24	1248 (29.1)	
25–29	2262 (52.7)	
Sex		
Male	2208 (51.4)	
Female	2088 (48.6)	
Time period		
Early (2010–2014)	2393 (55.7)	
Late (2015–2018)	1903 (44.3)	
Neighborhood income quintile		
Rural	426 (9.9)	
Urban Q1 (lowest)	733 (17.1)	
Urban Q2	797 (18.6)	
Urban Q3	756 (17.6)	
Urban Q4	770 (18.0)	
Urban Q5 (highest)	802 (18.7)	
Region		
Central	1328 (30.9)	
East	948 (22.1)	
North	293 (6.8)	
Toronto	443 (10.3)	
West	1282 (29.9)	
Cancer type		
Hematologic	1385 (32.2)	
Melanoma	238 (5.5)	
CNS	260 (6.1)	
Sarcoma	209 (4.9)	
Testicular/Ovarian	945 (22.0)	
Breast	331 (7.7)	
Colorectal	158 (3.7)	
Thyroid	196 (4.6)	
Other	574 (13.4)	
Chemotherapy within first year		
Yes	2415 (56.2)	
No	1881 (43.8)	
Radiotherapy within first year		
Yes	1481 (34.5)	
No	2815 (65.5)	
Cancer surgery within first year		
Yes	2553 (59.4)	
No	1743 (40.6%)	

Abbreviations: CNS, central nervous system; IQR, interquartile range; *N*, number.

### 3.1 Symptom prevalence, severity, and trajectory

**FIGURE 1:**
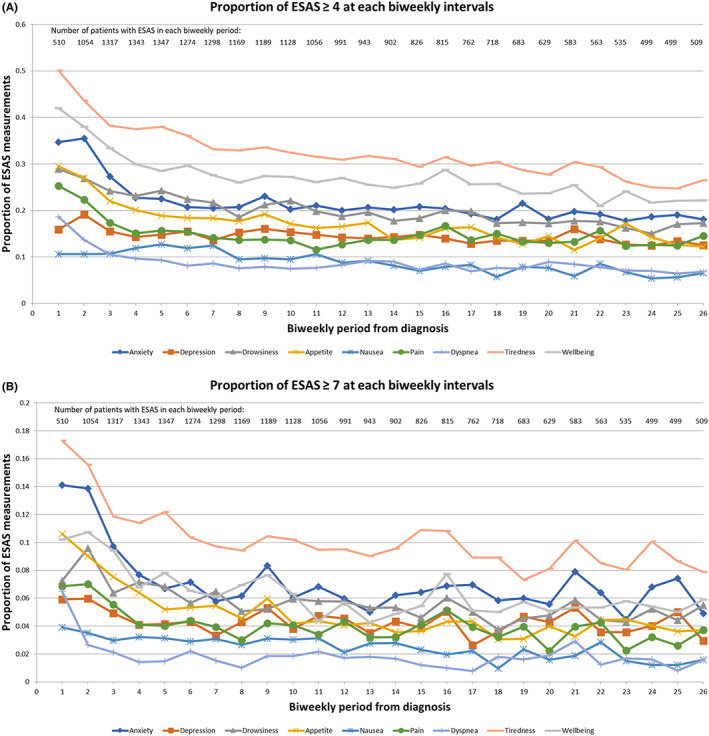
Proportion of AYA with moderate or severe symptoms by biweekly period. (A) Proportion of AYA with symptom scores ≥4 (moderate or severe). (B) Proportion of AYA with symptom scores ≥7 (severe). *Note that the y‐axis scale is not identical between the two parts of the figure.

The percentage of AYA who reported moderate or severe symptom burden at least once in the first year following diagnosis is shown in Table S2. Overall, AYA were most likely to report moderate or severe tiredness (59% and 27%), anxiety (44% and 19%), and impact on overall well‐being (52% and 20%). They were least likely to report dyspnea (23% and 6%) and nausea (24% and 9%). Prevalence of most symptoms decreased initially after diagnosis, but then plateaued (Figure 1A,B).

**FIGURE 2:**
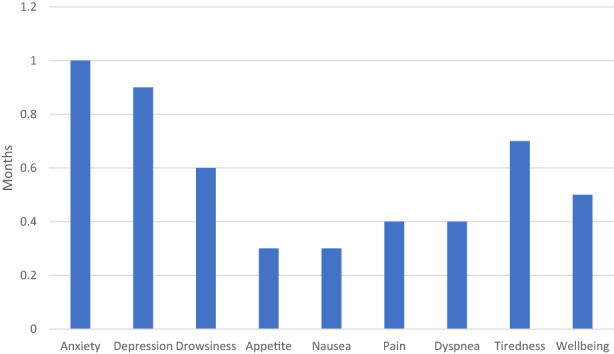
Mean sojourn time spent in the severe state, by symptom type.

For AYA reporting severe (ESAS score ≥7) scores in one or more symptoms, estimated mean length of time in this severe state is shown in Figure 2, stratified by symptom type. The longest estimated mean sojourn times were seen in patients with anxiety (1 month), depression (0.9 months), and tiredness (0.7 months). The shortest mean sojourn time was seen in patients with severe nausea and loss of appetite (0.3 months each). Relative rates of improvement vs. worsening are shown in Table 2. Across symptom type, for AYA experiencing moderate symptoms, the rate (speed/intensity) of improving was two to seven times higher than the rate of worsening (i.e., reaching the severe state). For most symptoms, AYA with scores of 2 or 3 also had a higher rate of improving vs. worsening. In general, the risk of subsequent death increased with severity of a given reported symptom (Table S3 and Table 3). The subsequent risk of death was particularly high in patients with severe dyspnea (9.0% within 6 months), pain (8.0%), and drowsiness (7.5%).

**TABLE 2:** Relative rate of worsening or improving symptoms by symptom state.

Symptom state (ESAS score)	Anxiety	Depression	Drowsiness	Appetite	Nausea	Pain	Dyspnea	Tiredness	Well‐being
1	↑ 64.7	↑ 4.7	↑ 9.5	↑ 7.1	↑ 46.5	↑ 4.5	↑ 3.1	↑ 14.9	↑ 2.7
2	↓ 36.9	–	–	↓ 1.9	↓ 24.6	–	–	↓ 4.2	↑ 2.0
3	–	↓ 2.6	↓ 5.3	↓ 3.2	–	↓ 7.5	↓ 4.4	↓ 2.4	↓ 3.5
Moderate (4–6)	↓ 5.3	↓ 3.6	↓ 2.7	↓ 2.1	↓ 7.6	↓ 2.4	↓ 3.9	↓ 2.3	↓ 2.3

*Note*: Relative rates depicted in red and with an upgoing arrow (↑) denote increased rates of worsening symptoms (relative to improving) while relative rates depicted in blue and with a down‐going arrow (↓) indicate increased rates of improving symptoms (relative to worsening). As an example, for AYA with moderate depression, the rate of experiencing an improvement in depression was 3.6 times higher than the rate of experiencing worsening depression. For AYAs with pain score of 1, the rate of experiencing worsening pain was 4.5 times higher than the rate of experiencing improving pain. Cells with a dash “–” indicate a relative rate of 1.0 or with a confidence interval that includes 1.0. For anxiety and appetite, a non‐positive Hessian matrix meant that standard errors could not be produced.

**TABLE 3:** Risk (probability) of subsequent death within 6 months associated with each symptom state.

	Anxiety	Depression	Drowsiness	Appetite	Nausea	Pain	Dyspnea	Tiredness	Well‐being
0	2.6	2.6 (2.5–5.4)	2.4 (2.3–4.9)	2.5	2.8 (2.4–3.1)	2.4 (2.3–5.1)	2.6 (2.5–6.8)	2.2 (2.17–5.2)	2.2 (2.1–4.4)
1	3.4	3.7 (3.6–8.9)	3.4 (3.2–6.9)	3.6	4.0 (3.5–4.5)	3.5 (3.3–7.1)	3.8 (3.6–11.0)	2.9 (2.8–6.6)	3.0 (2.9–6.1)
2	3.4	3.9 (3.8–9.6)	3.5 (3.3–7.0)	3.8	4.0 (3.5–4.6)	3.7 (3.6–7.7)	4.2 (3.9–12.5)	3.0 (2.9–6.7)	3.5 (3.3–7.0)
3	3.8	4.3 (4.1–10.3)	3.7 (3.5–7.1)	4.0	4.5 (3.8–5.3)	3.9 (3.7–7.9)	4.5 (4.3–13.8)	3.3 (3.1–7.3)	3.7 (3.6–7.5)
Moderate	4.0	5.2 (4.9–10.8)	4.5 (4.5–8.3)	4.8	4.7 (4.0–5.7)	5.4 (5.1–10.0)	6.0 (5.6–15.9)	4.1 (3.9–7.8)	4.7 (4.4–8.5)
Severe	4.3	5.7 (5.1–14.5)	7.5 (6.7–11.5)	5.9	5.8 (4.5–7.2)	8.0 (7.1–12.8)	9.0 (7.6–22.6)	5.9 (5.3–9.4)	5.5 (4.9–10.9)

*Note*: Greater risk (probability) of death is depicted in darker shades of red. For anxiety and appetite, a non‐positive Hessian matrix meant that standard errors could not be produced.

### 3.2 Predictors of high symptom burden

Variables associated with either moderate/severe (score ≥4) or severe (score ≥7) symptoms are shown in Table S4 and Table 4. Females were more likely to report severe symptoms than males [e.g., for nausea, adjusted hazard ratio (aHR) 1.7, 95th confidence interval (95% CI): 1.3–2.2]. AYA in the poorest urban neighborhoods were also more likely to report symptoms than those in the wealthiest urban areas. For example, these AYA were nearly twice as likely to experience severe depression (aHR: 2.0, 95% CI: 1.4–2.8), pain (aHR: 2.0, 95% CI: 1.4–2.7), or dyspnea (aHR: 2.0, 95% CI: 1.3–3.0). Though not consistent across symptom type, AYA living in Northern Ontario were more likely to report worse symptoms compared to those in Toronto, particularly of anxiety (aHR: 1.7, 95% CI: 1.2–2.6), lack of appetite (aHR: 1.7, 95% CI: 1.1–2.4), nausea (aHR: 1.9, 95% CI: 1.0–3.4), and pain (aHR: 1.8, 95% CI: 1.1–2.8). In contrast, apart from depression, rural AYA were not at increased risk of reporting moderate/severe or severe symptoms. Consistent patterns by cancer type were not seen.

**TABLE 4:** Predictors of ESAS score of greater or equal than seven (severe) among adolescents and young adults with cancer by symptom type.

	Anxiety	Depression	Drowsiness	Appetite	Nausea	Pain	Dyspnea	Tiredness	Well‐being
Age (years)	1.22 (0.96–1.55)	1.04 (0.77–1.41)	0.83 (0.64–1.08)	0.99 (0.76–1.27)	0.94 (0.67–1.32)	1.09 (0.81–1.45)	0.87 (0.58–1.28)	0.98 (0.80–1.20)	1.02 (0.81–1.28)
Sex									
Male	Ref	Ref	Ref	Ref	Ref	Ref	Ref	Ref	Ref
Female	**1.81 (1.51–2.19)**	**1.49 (1.18–1.88)**	**1.40 (1.15–1.70)**	**1.38 (1.15–1.65)**	**1.71 (1.33–2.19)**	**1.38 (1.11–1.72)**	1.11 (0.83–1.49)	**1.63 (1.40–1.89)**	**1.32 (1.12–1.56)**
Time period									
Early (2010–2014)	Ref	Ref	Ref	Ref	Ref	Ref	Ref	Ref	Ref
Late (2015–2018)	**1.20 (1.02–1.41)**	1.22 (0.98–1.51)	1.00 (0.84–1.19)	**0.71 (0.60–0.84)**	0.72 (0.57–0.92)	0.96 (0.78–1.17)	0.81 (0.62–1.07)	0.96 (0.84–1.10)	0.92 (0.79–1.08)
Neighborhood income quintile									
Rural	1.28 (0.91–1.80)	**1.70 (1.08–2.69)**	0.86 (0.59–1.26)	1.07 (0.76–1.51)	0.80 (0.50–1.28)	1.15 (0.73–1.81)	0.74 (0.41–1.36)	0.84 (0.63–1.12)	1.16 (0.83–1.62)
Urban Q1 (lowest)	**1.38 (1.04–1.83)**	**1.95 (1.37–2.78)**	1.27 (0.95–1.69)	**1.48 (1.11–1.97)**	1.25 (0.87–1.81)	**1.94 (1.39–2.70)**	**1.96 (1.27–3.02)**	**1.31 (1.05–1.63)**	**1.41 (1.08–1.82)**
Urban Q2	1.31 (0.98–1.73)	**1.80 (1.25–2.59)**	1.20 (0.89–1.60)	**1.46 (1.10–1.94)**	1.07 (0.75–1.53)	**1.61 (1.15–2.25)**	1.09 (0.70–1.72)	1.07 (0.85–1.33)	1.16 (0.88–1.51)
Urban Q3	1.27 (0.96–1.68)	**1.65 (1.13–2.41)**	1.03 (0.75–1.40)	1.28 (0.94–1.74)	0.75 (0.50–1.12)	1.20 (0.83–1.72)	1.47 (0.95–2.27)	0.96 (0.76–1.21)	**1.37 (1.05–1.78)**
Urban Q4	1.12 (0.84–1.50)	**1.50 (1.02–2.20)**	1.06 (0.79–1.42)	1.26 (0.96–1.67)	0.77 (0.53–1.13)	1.33 (0.95–1.87)	1.11 (0.72–1.72)	0.94 (0.75–1.18)	1.27 (0.98–1.65)
Urban Q5 (highest)	Ref	Ref	Ref	Ref	Ref	Ref	Ref	Ref	Ref
Region									
Central	1.20 (0.88–1.64)	1.09 (0.75–1.58)	1.01 (0.74–1.39)	1.11 (0.81–1.52)	1.49 (0.91–2.42)	1.42 (0.99–2.03)	1.11 (0.66–1.88)	1.06 (0.83–1.36)	0.93 (0.72–1.21)
East	1.12 (0.80–1.56)	0.99 (0.66–1.49)	1.12 (0.80–1.56)	1.14 (0.83–1.58)	1.60 (0.97–2.64)	1.37 (0.94–2.00)	1.19 (0.69–2.06)	1.13 (0.87–1.47)	0.78 (0.59–1.04)
North	**1.73 (1.18–2.55)**	1.27 (0.77–2.11)	1.16 (0.75–1.78)	**1.66 (1.13–2.43)**	**1.89 (1.04–3.43)**	**1.75 (1.11–2.78)**	1.32 (0.69–2.51)	1.32 (0.95–1.85)	0.97 (0.68–1.38)
Toronto	Ref	Ref	Ref	Ref	Ref	Ref	Ref	Ref	Ref
West	1.26 (0.92–1.74)	1.02 (0.69–1.50)	1.05 (0.76–1.44)	1.13 (0.83–1.55)	1.43 (0.87–2.34)	**1.44 (1.00–2.06)**	1.13 (0.67–1.91)	1.09 (0.85–1.40)	0.76 (0.58–0.99)
Cancer type									
Hematologic	0.79 (0.59–1.07)	0.84 (0.56–1.27)	0.98 (0.69–1.39)	1.16 (0.80–1.67)	1.29 (0.80–2.09)	0.84 (0.58–1.23)	0.86 (0.50–1.47)	0.96 (0.74–1.27)	**0.68 (0.50–0.93)**
Melanoma	0.73 (0.47–1.15)	0.64 (0.33–1.23)	0.89 (0.50–1.59)	1.23 (0.72–2.12)	1.55 (0.78–3.10)	0.70 (0.38–1.29)	0.52 (0.19–1.44)	0.77 (0.50–1.18)	**0.60 (0.37–0.97)**
CNS	0.92 (0.61–1.40)	0.90 (0.52–1.56)	1.23 (0.76–2.00)	1.41 (0.89–2.21)	**1.92 (1.07–3.44)**	0.98 (0.58–1.66)	0.66 (0.32–1.36)	1.21 (0.85–1.72)	1.07 (0.72–1.60)
Sarcoma	1.18 (0.74–1.90)	1.37 (0.79–2.40)	1.39 (0.85–2.28)	**1.86 (1.09–3.17)**	**2.47 (1.25–4.88)**	**2.98 (1.87–4.76)**	1.23 (0.60–2.51)	1.36 (0.93–2.00)	1.24 (0.81–1.92)
Testicular/Ovarian	0.71 (0.50–1.01)	0.66 (0.42–1.05)	**0.64 (0.43–0.96)**	1.23 (0.82–1.84)	1.48 (0.87–2.52)	0.69 (0.44–1.09)	0.64 (0.35–1.19)	0.76 (0.56–1.04)	0.79 (0.56–1.12)
Breast	Ref	Ref	Ref	Ref	Ref	Ref	Ref	Ref	Ref
Colorectal	1.06 (0.68–1.65)	**1.93 (1.13–3.30)**	1.54 (0.91–2.58)	**1.84 (1.09–3.10)**	1.38 (0.68–2.84)	**2.01 (1.17–3.45)**	1.23 (0.53–2.84)	1.39 (0.92–2.10)	1.21 (0.79–1.86)
Thyroid	1.03 (0.67–1.58)	1.32 (0.75–2.33)	**2.27 (1.42–3.62)**	1.13 (0.63–2.02)	0.82 (0.30–2.25)	0.46 (0.20–1.06)	1.48 (0.66–3.30)	**2.33 (1.61–3.35)**	1.33 (0.85–2.09)
Other	1.19 (0.87–1.63)	1.51 (0.99–2.31)	**1.67 (1.16–2.41)**	**2.38 (1.63–3.47)**	**3.23 (2.00–5.19)**	**2.01 (1.37–2.95)**	1.30 (0.73–2.31)	**1.43 (1.07–1.91)**	1.37 (0.99–1.91)
Cancer surgery									
Yes	0.90 (0.78–1.04)	0.94 (0.78–1.15)	0.92 (0.78–1.08)	0.98 (0.81–1.18)	0.90 (0.68–1.18)	0.97 (0.80–1.17)	**0.64 (0.46–0.90)**	**0.82 (0.72–0.94)**	0.90 (0.77–1.05)
No	Ref	Ref	Ref	Ref	Ref	Ref	Ref	Ref	Ref
Radiotherapy									
Yes	**0.71 (0.60–0.84)**	0.87 (0.70–1.09)	**1.26 (1.08–1.48)**	**1.53 (1.26–1.87)**	**1.38 (1.07–1.79)**	**1.31 (1.07–1.61)**	1.05 (0.76–1.44)	**1.30 (1.15–1.48)**	1.15 (0.97–1.36)
No	Ref	Ref	Ref	Ref	Ref	Ref	Ref	Ref	Ref
Chemotherapy									
Yes	**0.67 (0.59–0.75)**	0.82 (0.71–0.96)	1.09 (0.95–1.25)	0.86 (0.73–1.01)	**1.35 (1.11–1.64)**	**0.84 (0.71–1.00)**	0.78 (0.60–1.00)	1.01 (0.91–1.12)	**0.86 (0.76–0.98)**
No	Ref	Ref	Ref	Ref	Ref	Ref	Ref	Ref	Ref

*Note*: Bolded values represent statistically significant associations at *p* < 0.05.

Sensitivity analyses assuming that all patients' initial ESAS state was 0, or excluding those with only one ESAS score in the first year of treatment did not significantly change the results.

## 4 DISCUSSION

In this population‐based study of patient‐reported symptoms among AYA, we found significant initial symptom burden, particularly related to fatigue and anxiety. We also found that risk of subsequent death increased with symptom severity, and that particular subgroups of AYA (e.g., those in poor urban neighborhoods) were more likely to experience severe symptoms.

The most prevalent symptom in our cohort was fatigue, with 59% of the cohort reporting at least moderate fatigue during the first year after diagnosis and 27% reporting severe fatigue. Other studies, including a recent systematic review, have found cancer‐related fatigue to be prevalent and long‐lasting among AYA.^32^, ^33^ Indeed, in our cohort 27% of patients reported at least moderate fatigue even 1 year after diagnosis. Interventions such as programs of structured physical activity are commonly recommended by clinicians, though their effectiveness is not clear in AYA populations.^33^, ^34^, ^35^ Similarly, the prevalence of anxiety in our cohort was high (44% at least moderate, 19% severe). Patients reporting severe anxiety also had the longest duration in this state of any severe symptom, with a mean sojourn time of 1 month. Previous studies have also found high rates of anxiety among AYA with cancer.^36^, ^37^ To a greater extent than for cancer‐related fatigue, interventions for anxiety during cancer treatment, both pharmacologic, such as selective serotonin reuptake inhibitors (SSRIs), and non‐pharmacologic, such as cognitive‐behavioral therapy, have proven efficacy.^38^ However, barriers remain, including inadequate screening, lack of psychosocial and psychiatric resources, including community‐based patient organizations, and discomfort among oncologists with prescribing psychiatric medications.^39^ Our results suggest that interventions addressing these barriers to the uptake of supportive and psychosocial care interventions targeting anxiety, and research identifying effective supportive and psychosocial care interventions for cancer‐related fatigue will have a major impact in decreasing overall symptom burden among AYA with cancer.

Despite this, it is encouraging that across the symptoms measured, AYA with moderate symptoms were substantially more likely to experience symptom improvement versus deterioration. This finding however warrants caution, as we could not distinguish between improvements due to interventions prescribed by healthcare providers versus resolution as part of the natural course of the treatment. As seen by other authors, patients with mild symptoms were more likely to experience worsening rather than resolution.^40^ For example, AYA reporting mild nausea (ESAS score 1) were 40 times more likely to subsequently rate their nausea worse. Whether oncology care providers perceive mild symptoms as not needing intervention, or are prescribing ineffective interventions is unknown. A recent study of children and AYA with cancer found that only a third received guideline‐concordant care for prevention of acute chemotherapy‐induced nausea and vomiting (CINV).^41^ Ensuring that AYA experiencing mild symptoms receive adequate supportive care interventions to prevent further escalation should improve overall symptom burden.

Appropriate integration of palliative care into AYA cancer care remains challenging given distinct developmental and psychosocial needs in this population.^42^, ^43^, ^44^ Despite AYA being at high risk of intensive end‐of‐life care and the effectiveness of palliative care in decreasing this risk, palliative care referrals are limited and often late.^45^, ^46^ In our cohort, the risk of death within 6 months of a rating of a severe symptom ranged from 4.3% to 9.0%, with the highest risks associated with severe dyspnea or pain. Several authors have advocated for the use of “automatic triggers” for palliative care referrals such as unplanned hospitalizations or ICU admissions.^47^, ^48^, ^49^ Our results suggest that automatic palliative care integration based on severe symptoms, as measured by routine self‐assessments, may aid in symptom management and help initiate discussions surrounding advanced care planning. Importantly however, triggers based on severe symptoms may best function as a safety net for patients in whom earlier opportunities for palliative care involvement were missed.

We also found substantial inequities in symptom burden within our cohort. AYA residing in the poorest urban neighborhoods were more likely to experience moderate and severe symptoms compared to those in the wealthiest areas. This is of particular concern as previous work found that these same AYA were also less likely to complete ESAS scores at all.^26^ Similarly, AYA in Northern Ontario experienced more severe symptoms than those in urban Toronto. Multiple mechanisms underlying these disparities are possible. Fewer supportive and psychosocial care resources may be available in rural and remote areas or within hospitals serving low‐income populations. Second, patients living in areas of relative disadvantage, particularly areas at distance from oncology centers, may be substantially sicker before seeing their provider. Alternatively, oncologists' likelihood of intervening for a given level of symptoms may depend on patient factors including socioeconomic status, race, or ethnicity.^50^, ^51^, ^52^, ^53^, ^54^, ^55^, ^56^ For example, Check et al^55^ found both racial and socioeconomic disparities in the use of aprepitant for CINV prevention among women with breast cancer. Future studies should explore these mechanisms and possible interventions aimed at their mitigation. Our results indicate that a focus on supportive and psychosocial care is key for improving AYA cancer care, including focusing on those symptoms causing the greatest distress and ensuring equitable access across different subpopulations of AYA.

Strengths of this study include population‐wide symptom assessment scores, linkage to other health services databases, and appropriate statistical models for longitudinal symptom data. In addition to those mentioned above, several limitations merit note. First, 21% of our cohort was not ESAS screened during a year of their cancer diagnosis. Past work has shown that these patients are more likely to live in low‐income urban neighborhoods or to have hematologic malignancies.^26^ The symptom disparities we describe may thus be underestimated. On the other hand, patients with higher symptom burden may be more likely to complete ESAS questionnaires, resulting in an overestimate of the prevalence severe symptoms. Second, our cohort represents a heterogeneous mix of cancers. Though we accounted for malignancy type, our results may not be generalizable to specific subpopulations. Future studies will examine symptom burden among specific AYA cancers. Similarly, this heterogeneity extends to treatment‐related covariates. For example, antineoplastic regimens grouped under “having received chemotherapy” would have encompassed both intensive myelosuppressive chemotherapy as well as low‐intensity maintenance regimens. Fourth, race and/or ethnicity was not routinely collected. Fifth, our results may not be generalizable to other jurisdictions, particularly those without universal health insurance systems, or to older AYA aged 30–39 years. Sixth, our analyses were restricted to the first year after cancer diagnosis; symptom burden during survivorship is a concern but was not our focus. Finally, ESAS symptoms may not include some relevant to AYA populations, such as sexual function, impact on relationships, or changes in appearance.^10^, ^57^ Population‐based studies of AYA symptoms as determined by AYA‐specific measures would be of significant interest.^57^, ^58^

In conclusion, AYA with cancer experience substantial symptom burden at diagnosis which decreases over time. Cancer‐related fatigue and anxiety were the most prevalent and were among symptoms remaining in the severe state the longest. AYA with potential disadvantage (e.g., in poor urban neighborhoods or Northern areas) were more likely to experience severe symptoms. Subsequent risk of death increased with increasing symptom severity; symptom burden may be an appropriate trigger for palliative care referral if not already involved. Future studies should examine the impact of supportive and psychosocial care interventions such as involvement of social work, other psychosocial personnel, community‐based AYA organizations, or palliative care.

### PRIOR PRESENTATIONS

This work was presented at the 54th Congress of the International Society for Paediatric Oncology (SIOP) in September 2022.

## AUTHOR CONTRIBUTIONS

**Sumit Gupta:** Conceptualization (equal); funding acquisition (equal); methodology (equal); writing – original draft (equal); writing – review and editing (equal). **Qing Li:** Formal analysis (lead); writing – review and editing (equal). **Paul Nathan:** Writing – review and editing (equal). **Norma D'Agostino:** Writing – review and editing (equal). **Nancy N. Baxter:** Writing – review and editing (equal). **Colleen Fox:** Writing – review and editing (equal). **Karine Chalifour:** Writing – review and editing (equal). **Natalie Coburn:** Conceptualization (equal); methodology (equal); writing – review and editing (equal). **Rinku Sutradhar:** Conceptualization (equal); methodology (equal); writing – review and editing (equal).

## FUNDING INFORMATION

This work was supported by a grant from a partnership between the Terry Fox Research Institute and the Canadian Institute for Health Research (grant number TFX‐168820).

## CONFLICT OF INTEREST STATEMENT

The authors declare no relevant conflicts of interests.

## Supporting information


Appendix S1.

## Data Availability

The data underlying this article cannot be shared due to Ontario privacy legislation regulating the use of Ontario personal health information.
