# Supplementary material for: Prevalence, severity, and predictors of symptom burden among adolescents and young adults with cancer
Source: Cancer Med. 2023 Mar 27;12(10):11773–85. doi: 10.1002/cam4.5837 (PMC10242332; doi:10.1002/cam4.5837)

Supplemental Figure 1. Multistate transition model


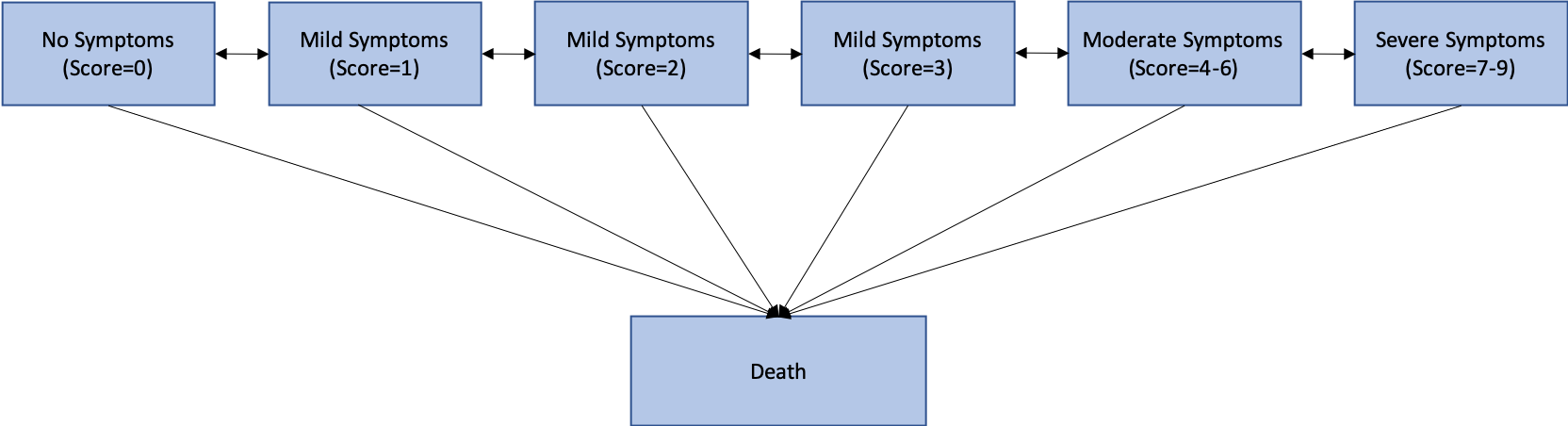


Supplemental Figure 2. Cohort meeting inclusion criteria


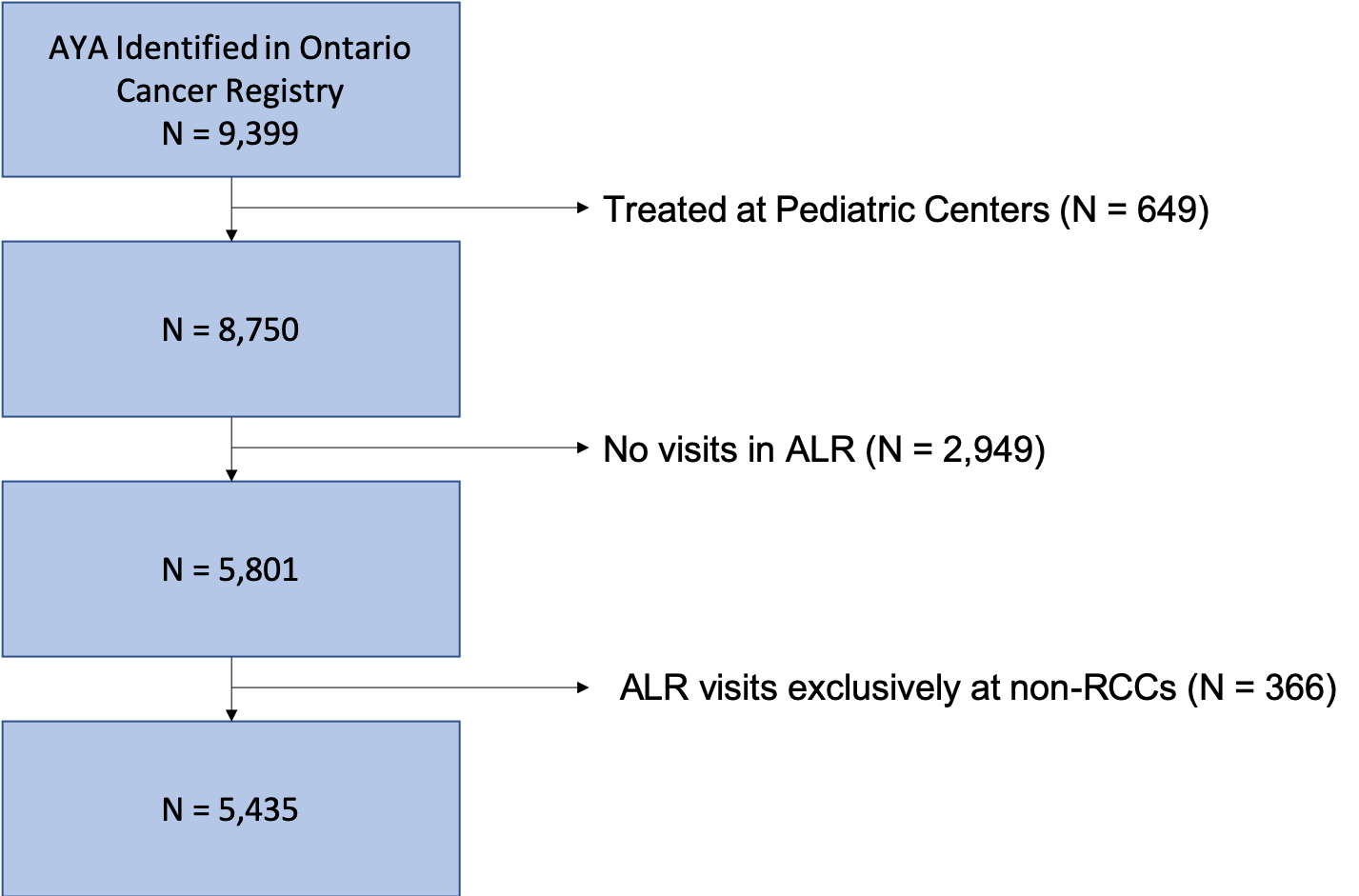


ALR – Activity Level Reporting database; AYA – Adolescent and young adult; RCC – Regional cancer center

Supplemental Table 1. Population-based health services databases used in this study

| **Database** | **Data Elements** | **Description** | **Initiation Year** |
| --- | --- | --- | --- |
| ALR | Cancer system therapies | Patient-level activity within the cancer system, focused on radiation therapy and systemic therapies, including chemotherapy. Also captures outpatient oncology visits | 2005 |
| DAD | Inpatient hospitalizations | One record per hospital admission including chart-abstracted demographic, clinical and outcome data | 1988 |
| NACRS/SDS | ED visits/Same day surgery | Demographic, clinical and disposition data | 2000 |
| OCR | Cancer diagnoses | One record per new cancer diagnosis | 1964 |
| OHIP | Physician claims | Claims for services billed by fee-for-service Ontario physicians. Physicians under alternative funding plans are also required to submit shadow claims, ensuring capture of nearly all physician encounters | 1991 |
| SMRD | Symptom scores | Patient-reported symptom burden | 2007 |

ALR – Activity Level Reporting; DAD – Discharge Abstract Database; ED – emergency department; NACRS – National Ambulatory Care Reporting System; OCR – Ontario Cancer Registry; OHIP – Ontario Health Insurance Plan Claims Database; SMRD – Symptom Management Reporting Database; SDS – Same Day Surgery

Supplemental Table 2. Percentage of AYA with at least one report of moderate or severe symptom burden in the first year following cancer diagnosis, by symptom type

|  | **Percentage of patients with ESAS ≥4**  **(i.e. moderate or severe)** | **Percentage of patients with ESAS ≥7**  **(i.e. severe)** |
| --- | --- | --- |
| Anxiety | 44 | 19 |
| Depression | 30 | 11 |
| Drowsiness | 42 | 16 |
| Appetite | 39 | 17 |
| Nausea | 24 | 9 |
| Pain | 34 | 13 |
| Dyspnea | 23 | 6 |
| Tiredness | 59 | 27 |
| Wellbeing | 52 | 20 |

Supplemental Table 3. Risk (probability) of subsequent death within one month associated with each symptom state.

|  | Anxiety | Depression | Drowsiness | Appetite | Nausea | Pain | Dyspnea | Tiredness | Wellbeing |
| --- | --- | --- | --- | --- | --- | --- | --- | --- | --- |
| 0 | 0.3 | 0.3 (0.2-0.5) | 0.2 (0.1-0.4) | 0.2 | 0.3 (0.2-0.3) | 0.1 (0.1-0.3) | 0.3 (0.2-0.5) | 0.1 (0.1-0.4) | 0.1 (0.1-0.3) |
| 1 | 0.7 | 0.7 (0.6-2.2) | 0.4 (0.4-1.5) | 0.7 | 1.0 (0.8-1.2) | 0.5 (0.5-1.3) | 0.7 (0.6-2.3) | 0.3 (0.3-1.2) | 0.4 (0.3-0.9) |
| 2 | 0.7 | 0.7 (0.6-2.8) | 0.5 (0.4-1.5) | 0.8 | 1.0 (0.9-1.3) | 0.7 (0.6-1.6) | 0.8 (0.8-2.9) | 0.3 (0.3-1.2) | 0.5 (0.4-1.4) |
| 3 | 0.8 | 0.9 (0.8-3.2) | 0.6 (0.5-1.7) | 1 | 1.4 (1.0-1.8) | 0.8 (0.7-1.8) | 1.0 (0.9-3.3) | 0.5 (0.4-1.5) | 0.6 (0.6-1.6) |
| Moderate | 0.9 | 1.4 (1.2-3.1) | 1.2 (1.0-2.2) | 1.4 | 1.6 (1.3-2.3) | 1.7 (1.6-3.2) | 2.0 (1.8-5.0) | 0.9 (0.8-1.8) | 1.2 (1.0-2.1) |
| Severe | 0.9 | 1.4 (0.9-4.8) | 3.2 (2.4-4.7) | 2.3 | 2.4 (1.7-4.1) | 3.8 (3.0-6.2) | 4.5 (3.1-10.9) | 2.1 (1.6-3.3) | 1.7 (1.2-4.2) |

Greater risk (probability) of death is depicted in darker shades of red

For anxiety and appetite, a non-positive Hessian matrix meant that standard errors could not be produced

Supplemental Table 4. Predictors of ESAS score of greater or equal than 4 (moderate or severe) among adolescents and young adults with cancer by symptom type

|  | Anxiety | Depression | Drowsiness | Appetite | Nausea | Pain | Dyspnea | Tiredness | Wellbeing |
| --- | --- | --- | --- | --- | --- | --- | --- | --- | --- |
| Age (years) | **1.18 (1.00-1.39)** | 1.06 (0.87-1.28) | 0.92 (0.78-1.08) | 0.88 (0.74-1.04) | 0.95 (0.77-1.18) | 1.01 (0.84-1.21) | 1.02 (0.81-1.28) | 1.05 (0.91-1.22) | 1.12 (0.96-1.30) |
| Sex |  |  |  |  |  |  |  |  |  |
| Male | Ref | Ref | Ref | Ref | Ref | Ref | Ref | Ref | Ref |
| Female | **1.82 (1.61-2.06)** | **1.49 (1.29-1.73)** | **1.22 (1.08-1.38)** | **1.37 (1.21-1.56)** | **1.31 (1.12-1.53)** | **1.24 (1.08-1.42)** | 1.09 (0.93-1.28) | **1.55 (1.39-1.73)** | **1.47 (1.31-1.64)** |
| Time period |  |  |  |  |  |  |  |  |  |
| Early (2010-2014) | Ref | Ref | Ref | Ref | Ref | Ref | Ref | Ref | Ref |
| Late (2015-2018) | 1.11 (0.99-1.24) | 1.12 (0.98-1.28) | 1.01 (0.90-1.13) | **0.74 (0.66-0.83)** | **0.78 (0.68-0.90)** | 0.95 (0.84-1.08) | 0.92 (0.79-1.07) | 0.95 (0.86-1.05) | 0.97 (0.87-1.07) |
| Neighborhood income quintile |  |  |  |  |  |  |  |  |  |
| Rural | 1.04 (0.83-1.30) | 1.05 (0.79-1.40) | 0.92 (0.73-1.16) | 0.91 (0.72-1.15) | 0.97 (0.72-1.30) | 1.22 (0.95-1.57) | 0.76 (0.55-1.03) | 1.05 (0.86-1.29) | 0.90 (0.73-1.12) |
| Urban Q1 (lowest) | 1.21 (1.00-1.45) | **1.51 (1.22-1.88)** | 1.14 (0.95-1.37) | **1.24 (1.03-1.50)** | **1.29 (1.01-1.63)** | **1.50 (1.21-1.85)** | **1.51 (1.19-1.92)** | **1.22 (1.03-1.44)** | **1.21 (1.02-1.44)** |
| Urban Q2 | 1.05 (0.87-1.27) | 1.13 (0.90-1.42) | 1.04 (0.87-1.25) | 1.09 (0.90-1.31) | 1.19 (0.94-1.51) | **1.39 (1.13-1.71)** | 1.13 (0.89-1.43) | 1.04 (0.88-1.22) | 1.00 (0.84-1.19) |
| Urban Q3 | 1.11 (0.92-1.34) | 1.22 (0.97-1.53) | 0.89 (0.74-1.08) | 1.01 (0.83-1.22) | 1.04 (0.82-1.32) | 1.21 (0.98-1.49) | 1.25 (0.98-1.60) | 0.99 (0.84-1.17) | 1.02 (0.85-1.21) |
| Urban Q4 | 0.98 (0.81-1.18) | 1.13 (0.90-1.42) | 0.96 (0.80-1.15) | 1.04 (0.86-1.25) | 1.04 (0.83-1.32) | 1.15 (0.93-1.41) | 0.96 (0.75-1.23) | 1.03 (0.87-1.20) | 1.08 (0.91-1.28) |
| Urban Q5 (highest) | Ref | Ref | Ref | Ref | Ref | Ref | Ref | Ref | Ref |
| Region |  |  |  |  |  |  |  |  |  |
| Central | 1.10 (0.89-1.35) | 1.02 (0.81-1.29) | 0.93 (0.76-1.14) | **1.24 (1.00-1.54)** | **1.39 (1.05-1.85)** | 1.22 (0.97-1.55) | 1.05 (0.79-1.39) | 0.90 (0.76-1.08) | 0.90 (0.75-1.09) |
| East | 1.17 (0.94-1.45) | 1.10 (0.86-1.41) | 1.03 (0.83-1.27) | **1.45 (1.16-1.82)** | **1.47 (1.10-1.98)** | 1.15 (0.90-1.47) | 1.21 (0.90-1.61) | 1.01 (0.84-1.21) | 0.88 (0.72-1.07) |
| North | 1.40 (1.07-1.84) | 1.08 (0.78-1.49) | 0.98 (0.74-1.29) | **1.72 (1.30-2.27)** | **1.61 (1.12-2.31)** | 1.25 (0.91-1.71) | 1.11 (0.76-1.61) | 0.94 (0.73-1.20) | 0.93 (0.72-1.20) |
| Toronto | Ref | Ref | Ref | Ref | Ref | Ref | Ref | Ref | Ref |
| West | 1.15 (0.94-1.42) | 1.08 (0.85-1.36) | 1.00 (0.82-1.22) | **1.34 (1.08-1.67)** | **1.39 (1.04-1.85)** | 1.23 (0.97-1.55) | 1.26 (0.96-1.65) | 0.93 (0.78-1.11) | 0.85 (0.71-1.03) |
| Cancer type |  |  |  |  |  |  |  |  |  |
| Hematologic | 0.87 (0.70-1.08) | 0.97 (0.75-1.26) | 1.03 (0.81-1.30) | 1.08 (0.85-1.36) | **1.48 (1.09-2.00)** | **0.77 (0.61-0.97)** | 1.23 (0.90-1.68) | 1.09 (0.90-1.34) | 0.93 (0.75-1.14) |
| Melanoma | 0.87 (0.64-1.19) | 0.74 (0.50-1.09) | 0.85 (0.59-1.21) | 1.09 (0.77-1.54) | 1.44 (0.93-2.24) | **0.63 (0.44-0.91)** | **0.51 (0.28-0.92)** | 0.77 (0.57-1.04) | **0.65 (0.48-0.89)** |
| CNS | 0.89 (0.66-1.19) | 1.11 (0.79-1.56) | 1.30 (0.96-1.75) | 1.16 (0.85-1.60) | **2.01 (1.35-2.99)** | 0.82 (0.60-1.13) | 0.88 (0.57-1.34) | 1.30 (1.00-1.71) | 1.17 (0.89-1.54) |
| Sarcoma | 1.20 (0.87-1.65) | 1.17 (0.81-1.68) | 1.27 (0.91-1.77) | **1.54 (1.10-2.15)** | **2.00 (1.30-3.10)** | **1.93 (1.42-2.64)** | 1.42 (0.93-2.16) | **1.34 (1.01-1.80)** | 1.30 (0.97-1.75) |
| Testicular/Ovarian | 0.90 (0.71-1.14) | 0.84 (0.63-1.11) | **0.77 (0.59-0.99)** | 0.97 (0.75-1.26) | **1.47 (1.04-2.07)** | 0.59 (0.46-0.77) | 0.85 (0.60-1.20) | 0.90 (0.72-1.12) | 0.80 (0.64-1.01) |
| Breast | Ref | Ref | Ref | Ref | Ref | Ref | Ref | Ref | Ref |
| Colorectal | **1.49 (1.07-2.08)** | **1.71 (1.18-2.48)** | 1.34 (0.94-1.92) | **1.50 (1.04-2.16)** | **1.91 (1.21-3.02)** | 1.19 (0.81-1.74) | 1.09 (0.66-1.78) | 1.32 (0.96-1.82) | 1.17 (0.85-1.62) |
| Thyroid | 1.23 (0.89-1.69) | 1.43 (0.98-2.08) | **2.02 (1.45-2.80)** | 1.02 (0.69-1.51) | **1.74 (1.06-2.86)** | **0.56 (0.37-0.84)** | 1.30 (0.79-2.14) | **2.07 (1.54-2.79)** | 1.11 (0.81-1.52) |
| Other | 1.19 (0.95-1.51) | **1.36 (1.03-1.79)** | 1.25 (0.97-1.62) | **1.70 (1.32-2.19)** | **2.87 (2.09-3.95)** | **1.47 (1.15-1.87)** | 1.29 (0.92-1.80) | **1.27 (1.02-1.58)** | **1.26 (1.01-1.58)** |
| Cancer Surgery |  |  |  |  |  |  |  |  |  |
| Yes | 0.97 (0.89-1.06) | 1.08 (0.98-1.20) | 0.93 (0.84-1.02) | 1.00 (0.89-1.12) | 0.97 (0.83-1.13) | **1.12 (1.00-1.25)** | **0.83 (0.71-0.98)** | 0.94 (0.86-1.02) | **1.10 (1.01-1.21)** |
| No | Ref | Ref | Ref | Ref | Ref | Ref | Ref | Ref | Ref |
| Radiotherapy |  |  |  |  |  |  |  |  |  |
| Yes | **0.78 (0.70-0.87)** | 1.02 (0.91-1.13) | **1.44 (1.30-1.59)** | **1.31 (1.16-1.48)** | **1.52 (1.30-1.78)** | **1.42 (1.27-1.59)** | 1.11 (0.95-1.28) | **1.37 (1.25-1.49)** | **1.15 (1.05-1.26)** |
| No | Ref | Ref | Ref | Ref | Ref | Ref | Ref | Ref | Ref |
| Chemotherapy |  |  |  |  |  |  |  |  |  |
| Yes | **0.68 (0.63-0.73)** | **0.92 (0.84-0.99)** | **1.12 (1.04-1.21)** | 0.98 (0.89-1.07) | **1.62 (1.44-1.81)** | **0.89 (0.81-0.98)** | 0.92 (0.82-1.03) | 1.04 (0.98-1.11) | 0.99 (0.93-1.06) |
| No | Ref | Ref | Ref | Ref | Ref | Ref | Ref | Ref | Ref |

Bolded values represent statistically significant associations at p<0.05

Appendix 1


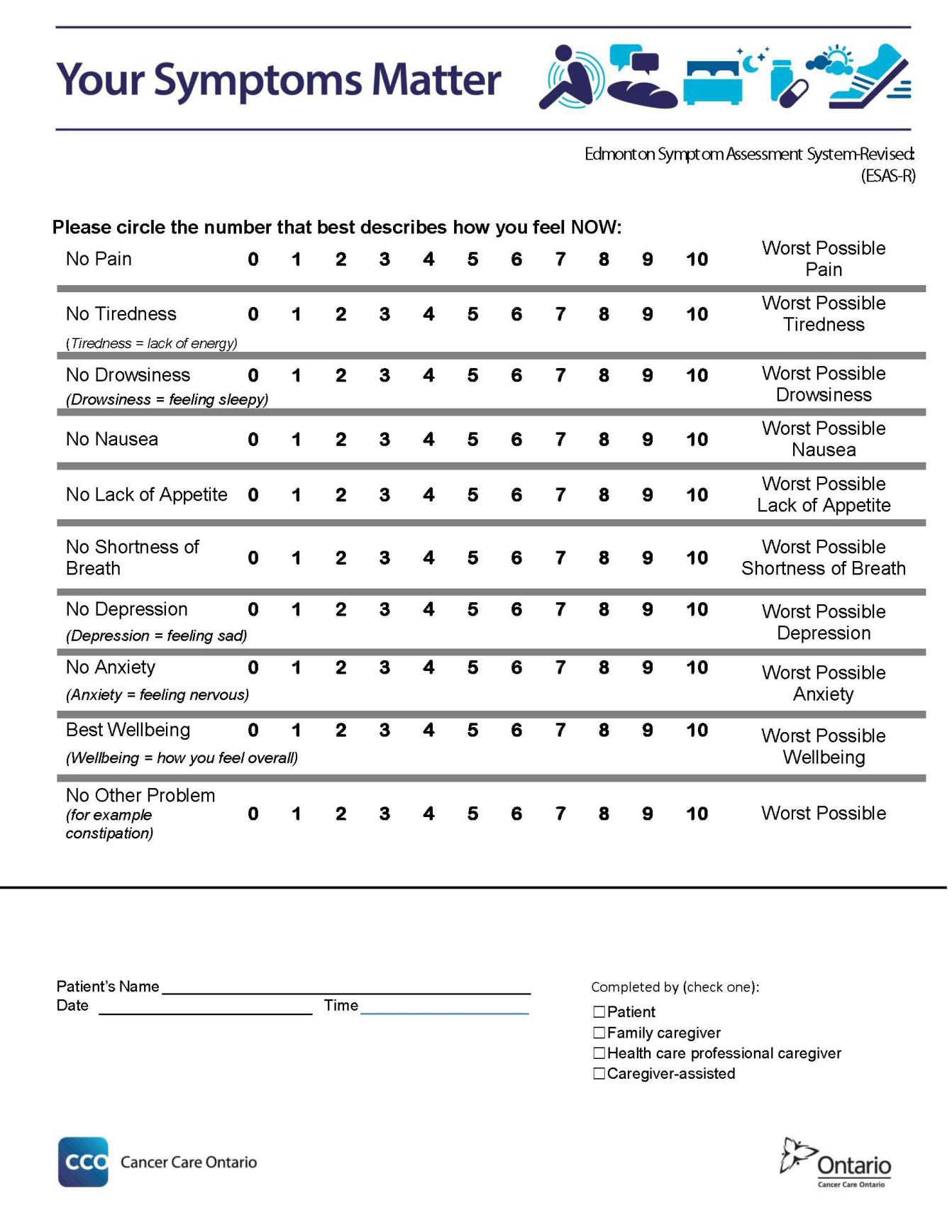

Supplement: Supplementary file 1 — Appendix S1. [file CAM4-12-11773-s001.docx]
